# Treatment of ipsilateral femoral neck and shaft fracture by augmented fixation via modified anterior approach: A case report

**DOI:** 10.1016/j.tcr.2022.100650

**Published:** 2022-04-27

**Authors:** Lin Qi, Wei Zhang, Hua Chen

**Affiliations:** a Chinese PLA Medical School, No.28 Fuxing Road, Haidian, Beijing 100853, China; b Department of Orthopaedics, the First Medical Center, Chinese PLA General Hospital, No.28 Fuxing Road, Haidian, Beijing 100853, China

**Keywords:** Femoral shaft fracture, Femoral neck fracture, Augmented fixation, Anterior approach

## Abstract

**Case:**

A 39-year-old man with obsolete fracture of the left femoral neck (AO/OTA31B2.3, Pauwels III) and segmental fracture of the ipsilateral shaft (AO/OTA32C2) caused by traffic accident was treated by a hybrid augmented fixation technique, long reconstruction intramedullary nail combined with medial anatomical buttress plate and poller screws. All procedures were carried out via the modified anterior approach with a good exposure of the fracture site.

**Conclusion:**

The hybrid augmented fixation technique via the modified anterior approach could improve fracture reduction and mechanical stability for ipsilateral femoral neck and shaft fractures.

## Introduction

Ipsilateral femoral neck and shaft fractures account for 1%–9% of femoral shaft fractures, typically high-energy injuries, and are common in young men [1], [2]. Femoral shaft fracture is typically comminuted and displaced in up to 33% of ipsilateral femoral neck and shaft fractures. Femoral neck fracture generally is basicervical and nondisplaced with the Pauwels angle usually more than 50 degrees, which is often missed or delayed diagnosis. Patients are usually associated with polytrauma [1], [2]. Attention should be given to the femoral neck fracture because of its complications, such as displacement, nonunion, malunion, and osteonecrosis, are challenging to manage and can lead to the need for arthroplasty in a young patient. In addition, the femoral reconstruction nail cannot provide enough biomechanical stability for the neck fracture [3]; augmented fixation has become the key to solving this problem. But the optimal implant choice is still controversial [1].

We present a patient with an obsolete and complicated ipsilateral femoral neck and shaft fracture treated by a new surgical strategy. A hybrid augmented fixation, which included a novel buttress plate for femoral neck fracture, was used. Meanwhile, the modified anterior approach was applied during operation to achieve fracture reduction under direct vision. The various difficulties encountered highlight the treatment challenges in patients with ipsilateral femoral neck and shaft fracture and provide valuable tips and techniques for future cases.

## Case report

**Fig. 1:**
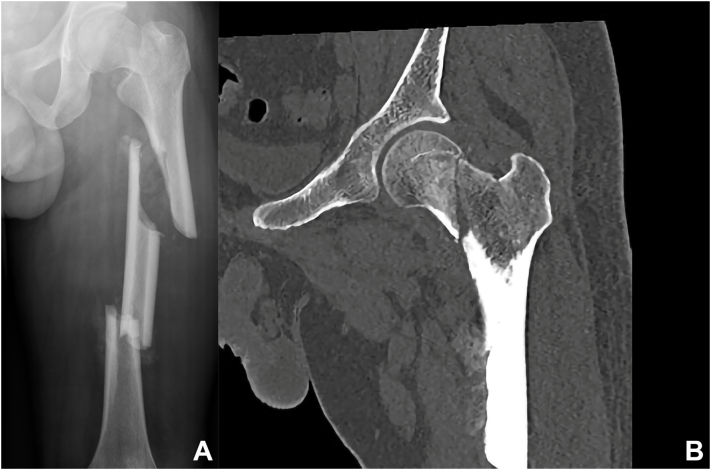
Preoperative imaging. A: X-ray showed ipsilateral left femoral neck and shaft fracture with obvious displacement. B: CT scan showed the fracture line of the left femoral neck is from subcapital to basicervical, and the Pauwels angle was more than 50 degrees.

A patient is a 39-year-old man with left limb pain and limited movement caused by a traffic accident for 35 days. Due to the severe abdominal injury caused by a traffic accident, the initial treatment focused on the abdominal trauma. Only tibial tubercle traction was given to the fracture of the lower extremity. The diagnosis of the injured extremity was that: complete transcervical fracture of the left femoral neck (AO/OTA31B2.3, Pauwels III), obsolete segmental fracture of the left femoral shaft (AO/OTA32C2) (Fig. 1).

Due to difficult reduction on complete fracture, open reduction and internal fixation were carried out under general anesthesia in July 2020. The patient was placed in the supine position on the OSI full fluoroscopic traction operation bed. The left tibial tubercle bone traction was connected with the lower limb traction system. The calf was rotated internally to keep the patella perpendicular to the ground, and the femoral shaft and femoral neck fractures were initially reduced by traction. The surface projection of the proximal and distal approach was determined in front of the thigh, the junction area of the isthmus and the non-isthmus of the proximal femoral shaft was taken as the boundary, the line between the anterior superior iliac spine and the lateral edge of the patella was taken as the incision surface projection in the proximal area, and the line between the anterior superior iliac spine and the medial edge of the patella was taken as the incision surface projection in the distal area [4], [5]. The location of the fracture site was determined by intraoperative C-arm fluoroscopy and marked horizontally on the body surface.

**Fig. 2:**
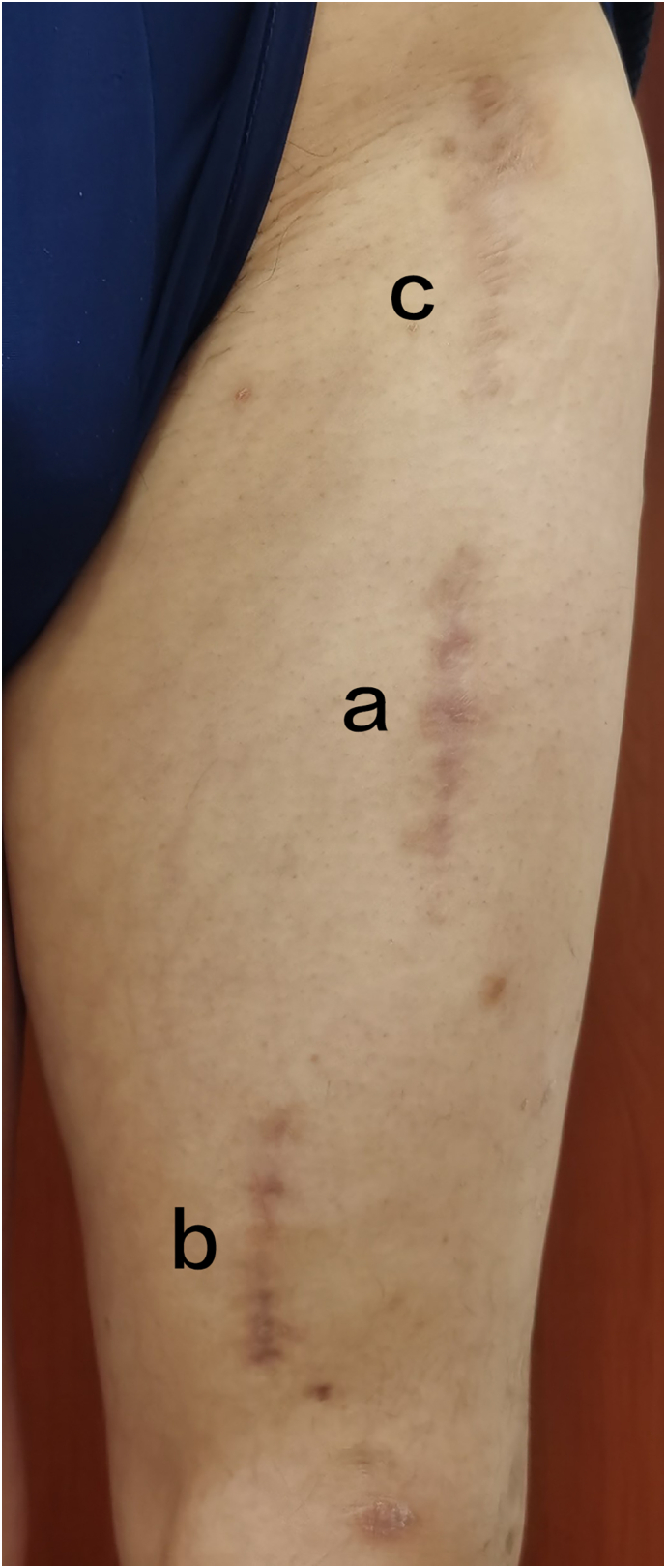
The skin incision healed well. (a) The anterolateral approach for the proximal femoral shaft fracture. (b) The anteromedial method for the distal femoral shaft fracture. (c) The anterior approach for the femoral neck fracture.

First of all, the femoral shaft fracture was reduced and fixed. The first step, according to the position of the surface projection of the proximal femoral shaft fracture line (anterolateral approach), a 6 cm longitudinal skin incision (Fig. 2-a) was performed, then open the subcutaneous and deep fascia to expose the rectus femoris, pull the rectus femoris to the medial side and split the vastus intermediu**s**, directly expose the proximal fracture site, which was reduced by Weber forceps under direct vision. Through the anteromedial approach (Fig. 2-b), the medial muscle space of the rectus femoris exposes and splits the vastus intermedius to the distal fracture site and achieves fracture reduction in a similar way as the proximal fracture site. The third step, after the femoral neck fracture was temporarily fixed with two percutaneous Kirschner wires, the Trigen Tan Fan (Simth&Nephew Ltd., Watford, UK) was placed, and the distal locking screws were firstly inserted after the position of the nail. The fracture reduction was confirmed by C-arm fluoroscopy. The next step is to augment the fixation of the non-isthmus region of the distal femoral shaft with two Poller screws to reduce the medullary cavity and decrease the nailing swing.

After the femoral shaft fracture was fixed, the femoral neck fracture was reduced and fixed. Through the anterior approach of the proximal femur (Fig. 2-c), the fracture site of the femoral neck was exposed, and two cephaloscrews were placed after confirming that the reduction was satisfactory. To counteract the high vertical shear force and compressive stress, the medial anatomical buttress plate (MABP) (ZhengTian Medical Instrument Co. Ltd., Tianjin, China) was used to fix the femoral neck fracture [6], [7].

After all, fractures were fixed, C-arm fluoroscopy confirmed again that the reduction of fractures and the position of internal fixation was satisfactory. Then the incision was closed layer by layer after flushing the incision and placing the drainage tube.

**Fig. 3:**
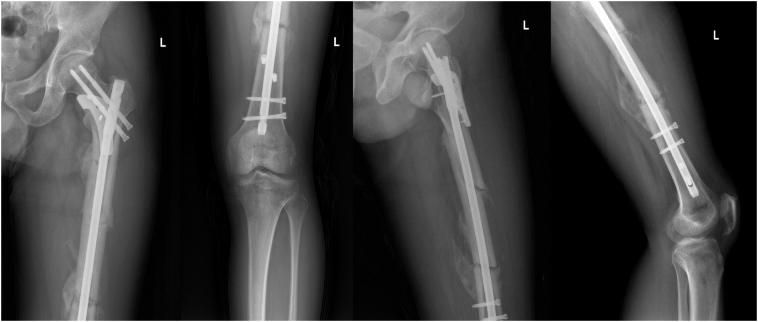
Radiographs were taken three months after surgery showed fracture healing.

**Fig. 4:**
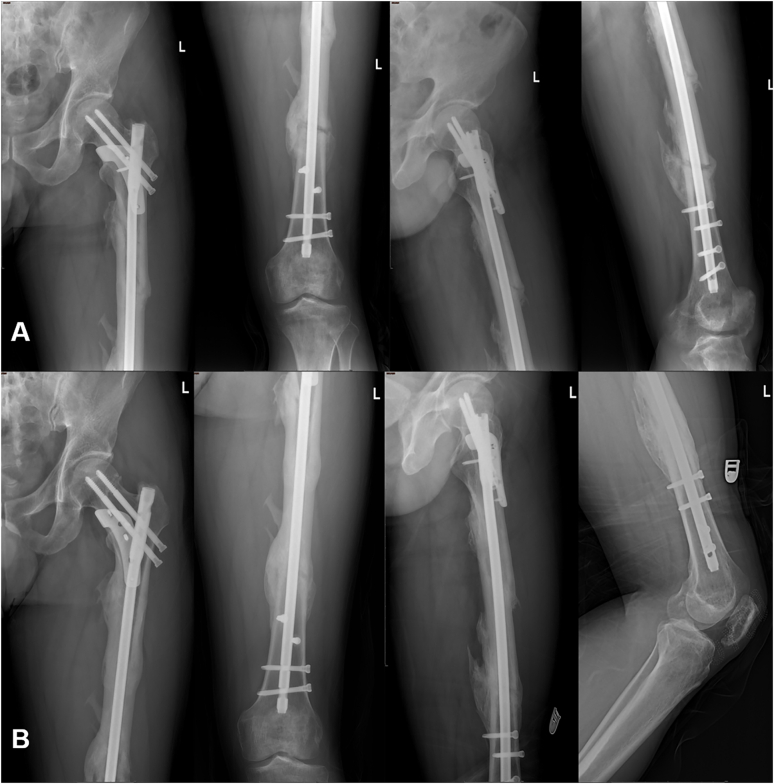
A: Radiographs at seven months after surgery. B: Radiographs at 17 months after surgery.

Due to reliable augmentation fixation for every fracture site, the patient immediately began the functional exercise postoperatively. Then, the patient was reviewed regularly in the outpatient clinic and instructed to increase the practical training intensity gradually. The patients were followed up at 3, 7, and 17 months after the operation; the radiographic follow-up results are shown in Fig. 3, Fig. 4, respectively; the function and quality of life evaluation results are shown in Table 1. Three months after the operation, the fracture healed, and he began the entire weight-bearing exercise. Finally, the patient had a good prognosis without complications.

**Table 1:** The evaluation results of the function and quality of life.

	3 months after the operation	7 months after the operation	17 months after the operation
OHS	32	44	47
LEFS	50	71	79
Pain VAS	2	1	1
PCS-12	39.96	42.47	53.13
MCS-12	56.32	49.47	59.87

OHS: Oxford Hip Score.

LEFS: Lower extremity functional scale.

Pain VAS: Pain Visual Analogue Scale.

PCS-12: Physical Component Score-12.

MCS-12: Mental Component Score-12.

## Discussion

Ipsilateral femoral neck and shaft fractures are relatively rare and challenging. Different approaches and fixation techniques have been used for this complex fracture, including closed and open surgery, single fixed system, or combined fixed system [2], [8]. Watson and Moed [6] reported eight femoral necks nonunion, and 75% (6/8) occurred in patients treated with a reconstruction nail. Ostrum et al. [2] wrote a multicenter series of 95 patients who underwent retrograde nailing for the femoral shaft fracture and either cannulated screws or a sliding hip screw for the femoral neck fracture. Femoral neck nonunion occurred in 5 cases, and femoral shaft nonunion occurred in 8 cases. Hung et al. [8] reported shaft nonunion occurred in 5 cases in a series of 47 patients in which the shaft was treated with plate fixation, and the proximal fracture was treated either with lag screws or a sliding hip screw. The possible reasons for the poor prognosis are as follows: (1) It is impossible to achieve a good reduction of angulation, rotation, and lateral displacement of the fracture end under closed conditions. (2) The traditional open reduction method seriously destroys the posterior end of the fracture end's blood supply. (3) The fracture end of Pauwels III femoral neck fracture has large shea forces, and the femoral reconstruction nail cannot provide enough biomechanical stability for the fracture end [3].

Considering the above problems and the patient's actual situation, we treated the patient using the following methods: (1) the modified anterior approach can expose the fracture site by crossing the intermuscular space without distributing essential nerves and blood vessels [4], [5]. (2) The hybrid augmented fixation technique: medial anatomical buttress plate (MABP) can make up for the mechanical defects of the reconstruction nail, resist the shear forces at the broken end, and increase the biomechanical stability of the broken end [9]. (3) Poller screws added to the distal non-isthmus of the femoral shaft can limit the movement of intramedullary nails in the broad medullary cavity and improve the biomechanical stability of the fixation structure. Finally, the patient was satisfied with his prognosis [10].

The delayed surgical treatment due to the polytrauma presents unique challenges for ipsilateral femoral neck and shaft fracture. More aggressive augmented fixation to improve construct stability with direct exposure for fracture site may be necessary. In particular, this novel buttress plate could effectively resist the vertical shear forces of femoral neck fracture. In summary, the hybrid augmented fixation technique via the modified anterior approach could achieve a good fracture reduction and mechanical stability for ipsilateral femoral neck and shaft fractures, finally leading to excellent clinical outcomes for the patient.

## Sources of funding

This work was supported by Preclinical Correlative Studies in Orthopedic Medical Device Products (YQCH201601) and Mechanical Study and Clinical Application of Triangular Stabilization System in the Treatment of Nonunion of Femoral Neck Fractures (XJS202107).

## Declaration of competing interest

None.

## Appendix A Supplementary data

United States Patent-MEDIAL BUTTRESS CONSTRUCT AND A FRACTURE FIXATION DEVICE FOR FEMORAL NECK (US 11,000,320 B2). Image 1
