# Supplementary material for: Treatment of ipsilateral femoral neck and shaft fracture by augmented fixation via modified anterior approach: A case report
Source: Trauma Case Rep. 2022 Apr 27;39:100650. doi: 10.1016/j.tcr.2022.100650 (PMC9092491; doi:10.1016/j.tcr.2022.100650)
Supplement: Supplementary file 1 — United States Patent-MEDIAL BUTTRESS CONSTRUCT AND A FRACTURE FIXATION DEVICE FOR FEMORAL NECK (US 11,000,320 B2). [file mmc1.pdf]

United States of America

To Promote the Progress of Science and Useful Arts

The Director

of the United States Patent and Trademark Office has received an application for a patent for a new and useful invention. The title and description of the invention are enclosed. The requirements of law have been complied with, and it has been determined that a patent on the invention shall be granted under the law.

Therefore, this United States

Patent

grants to the person(s) having title to this patent the right to exclude others from making, using, offering for sale, or selling the invention throughout the United States of America or importing the invention into the United States of America, and if the invention is a process, of the right to exclude others from using, offering for sale or selling throughout the United States of America, products made by that process, for the term set forth in 35 U.S.C. 154(a)(2) or (c)(1), subject to the payment of maintenance fees as provided by 35 U.S.C. 41(b). See the Maintenance Fee Notice on the inside of the cover.

Performing the Functions and Duties of the Under Secretary of Commerce for Intellectual Property and Director of the United States Patent and Trademark Office

Maintenance Fee Notice

If the application for this patent was filed on or after December 12, 1980, maintenance fees are due three years and six months, seven years and six months, and eleven years and six months after the date of this grant, or within a grace period of six months thereafter upon payment of a surcharge as provided by law. The amount, number and timing of the maintenance fees required may be changed by law or regulation. Unless payment of the applicable maintenance fee is received in the United States Patent and Trademark Office on or before the date the fee is due or within a grace period of six months thereafter, the patent will expire as of the end of such grace period.

Patent Term Notice

If the application for this patent was filed on or after June 8, 1995, the term of this patent begins on the date on which this patent issues and ends twenty years from the filing date of the application or, if the application contains a specific reference to an earlier filed application or applications under 35 U.S.C. 120, 121, 365(c), or 386(c), twenty years from the filing date of the earliest such application ("the twenty-year term"), subject to the payment of maintenance fees as provided by 35 U.S.C. 41(b), and any extension as provided by 35 U.S.C. 154(b) or 156 or any disclaimer under 35 U.S.C. 253.

If this application was filed prior to June 8, 1995, the term of this patent begins on the date on which this patent issues and ends on the later of seventeen years from the date of the grant of this patent or the twenty-year term set forth above for patents resulting from applications filed on or after June 8, 1995, subject to the payment of maintenance fees as provided by 35 U.S.C. 41(b) and any extension as provided by 35 U.S.C. 156 or any disclaimer under 35 U.S.C. 253.

US011000320B2

(12) **United States Patent**
Chen et al.

(10) **Patent No.: US 11,000,320 B2**
(45) **Date of Patent: May 11, 2021**

(54) **MEDIAL BUTTRESS CONSTRUCT AND A FRACTURE FIXATION DEVICE FOR FEMORAL NECK**

(71) Applicants: **Hua Chen**, Beijing (CN); **Peifu Tang**, Beijing (CN)

(72) Inventors: **Hua Chen**, Beijing (CN); **Peifu Tang**, Beijing (CN)

(73) Assignee: **Tianjin Zhengtian Medical Instrument Co., Ltd**, Tianjin (CN)

(*) Notice: Subject to any disclaimer, the term of this patent is extended or adjusted under 35 U.S.C. 154(b) by 18 days.

(21) Appl. No.: **16/075,166**

(22) PCT Filed: **Feb. 4, 2016**

(86) PCT No.: **PCT/CN2016/073541**

§ 371 (c)(1),
(2) Date: **Aug. 3, 2018**

(87) PCT Pub. No.: **WO2017/132952**

PCT Pub. Date: **Aug. 10, 2017**

(65) **Prior Publication Data**

US 2019/0038327 A1 Feb. 7, 2019

(51) **Int. Cl.**
A61B 17/74 (2006.01)
A61B 17/80 (2006.01)
(Continued)

(52) **U.S. Cl.**
CPC .... ***A61B 17/746*** (2013.01); ***A61B 17/74*** (2013.01); ***A61B 17/748*** (2013.01); (Continued)

(58) **Field of Classification Search**
CPC ... A61B 17/74; A61B 17/746; A61B 17/8061; A61B 17/82; A61B 17/1721; A61B 17/1725
See application file for complete search history.

(56) **References Cited**

U.S. PATENT DOCUMENTS

5,356,410 A * 10/1994 Pennig A61B 17/744 606/281
9,339,313 B1 * 5/2016 Powlan A61B 17/746
(Continued)

FOREIGN PATENT DOCUMENTS

CN 102429715 A 5/2012
CN 103860249 A 6/2014
(Continued)

OTHER PUBLICATIONS

International Search Report (and its English translation) and Written Opinion for PCT Application No. PCT/CN2016/073541 filed Feb. 4, 2016 (published as WO 2017/132952 on Aug. 10, 2017), dated Nov. 2, 2016, 9 pages.

Primary Examiner — Matthew J Lawson
(74) *Attorney, Agent, or Firm* — Harness, Dickey & Pierce, P.L.C.; Anthony G. Fussner

(57) **ABSTRACT**

A medial buttress construct for femoral neck includes a support part and an attachment fixing part fixedly connected to each other. The support part includes a structure matching an inferior surface of a proximal end of the femoral neck. The attachment fixing part includes structures simultaneously matching a front side surface of a distal end of the femoral neck and a front side surface of an upper end of femoral shaft. The attachment fixing part includes at least one opening for allowing a screw to pass through the structure matching the front side surface of the distal end of the femoral neck, and at least one opening for allowing a screw to pass through the structure matching the front side surface of the upper end of the femoral shaft. A fracture fixation device for femoral neck may include the medial buttress construct for femoral neck.

20 Claims, 5 Drawing Sheets

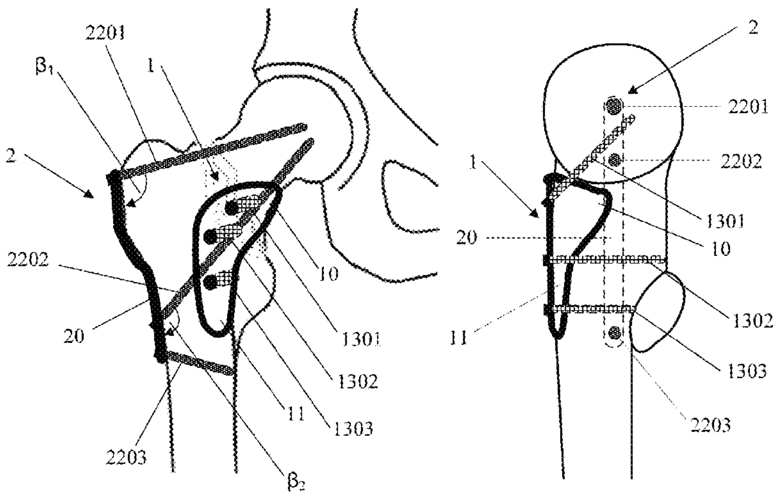

(51) **Int. Cl.**
A61B 17/17 (2006.01)
A61B 17/56 (2006.01)

(52) **U.S. Cl.**
CPC ***A61B 17/8061*** (2013.01); *A61B 17/1721* (2013.01); *A61B 17/1725* (2013.01); *A61B 2017/568* (2013.01)

(56) **References Cited**

U.S. PATENT DOCUMENTS

9,974,582	B1 *	5/2018	Anissian	A61B 17/8014
2005/0010226	A1 *	1/2005	Grady, Jr.	A61B 17/746 606/281
2008/0086137	A1	4/2008	Probe	
2012/0271366	A1 *	10/2012	Katrana	A61B 17/282 606/86 R
2013/0046307	A1 *	2/2013	Yang	A61B 17/744 606/64
2014/0378973	A1 *	12/2014	Mueckter	A61B 17/72 606/64

FOREIGN PATENT DOCUMENTS

CN	104224300	A	12/2014
CN	204863440	U	12/2015
CN	105520774	A	4/2016
CN	205514844	U	8/2016

* cited by examiner

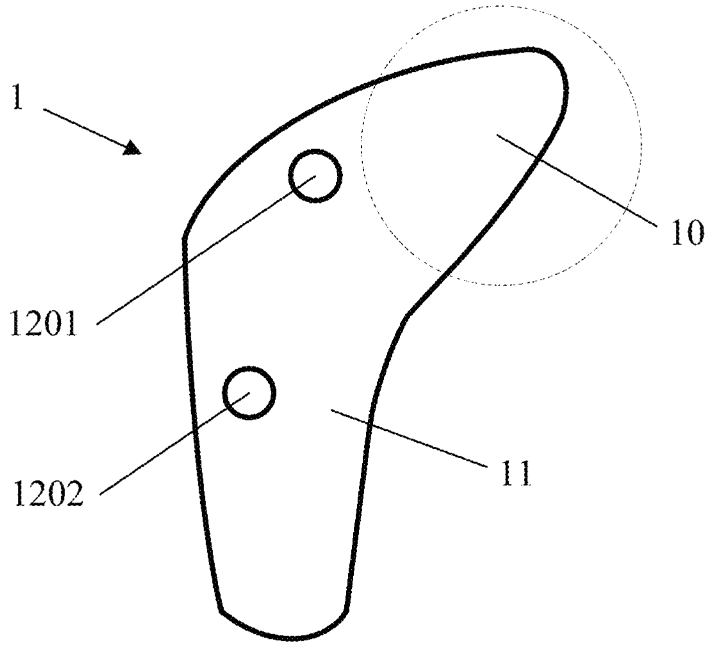

Figure 1

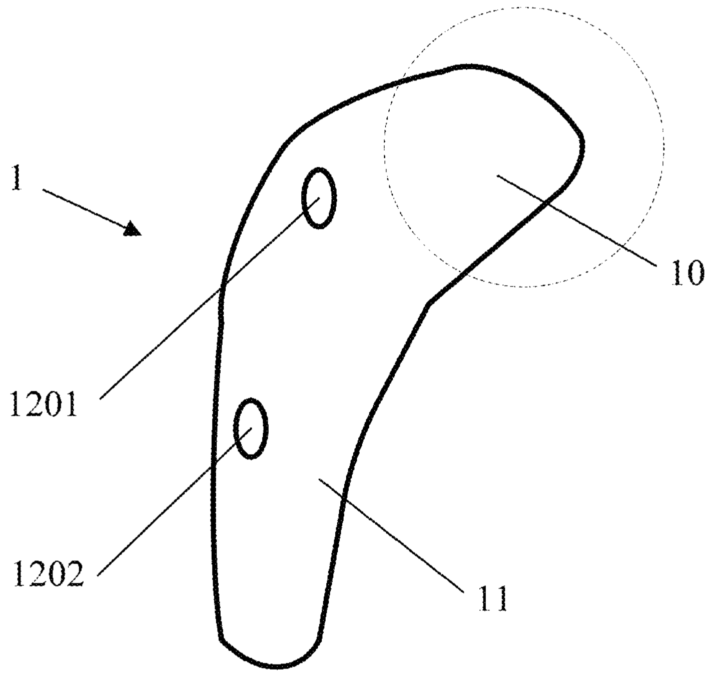

Figure 2

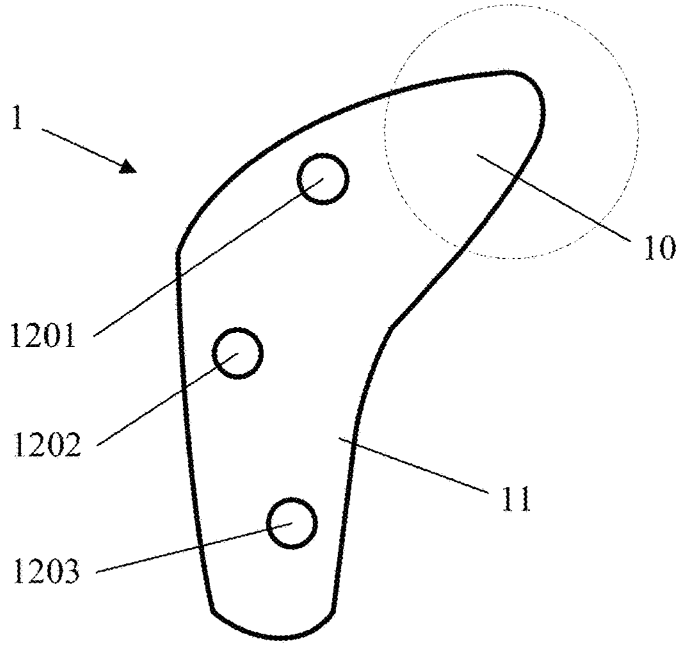

Figure 3

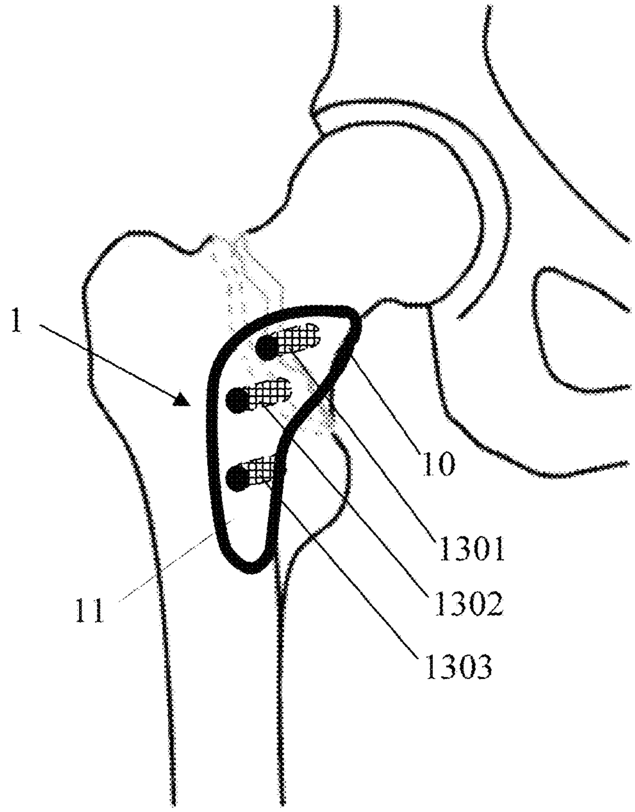

Figure 4

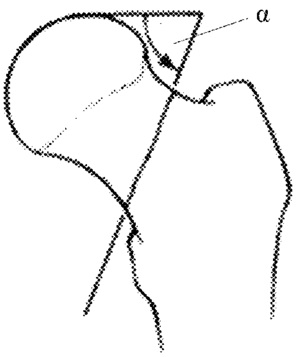

Figure 5

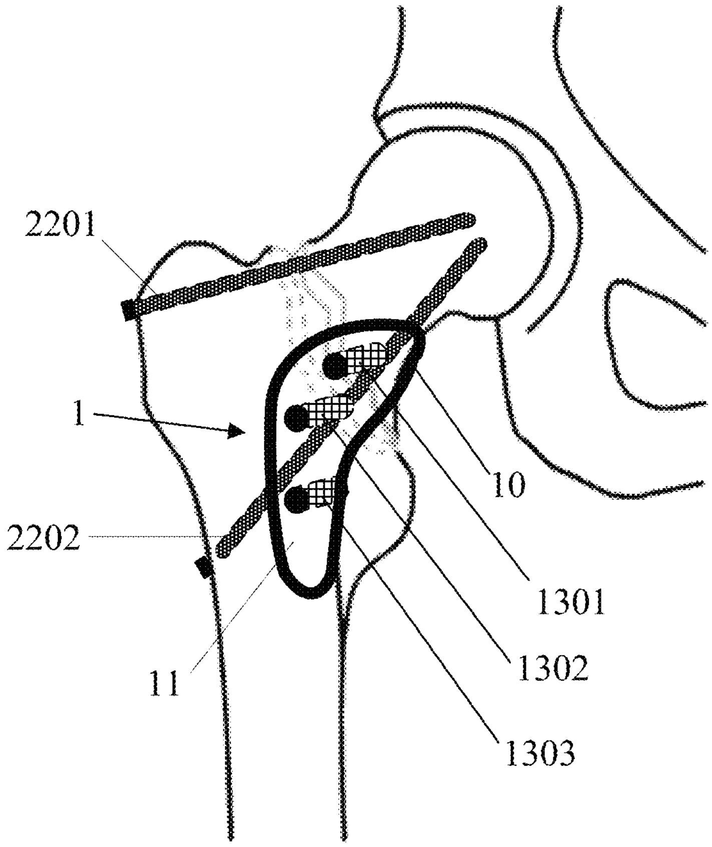

Figure 6

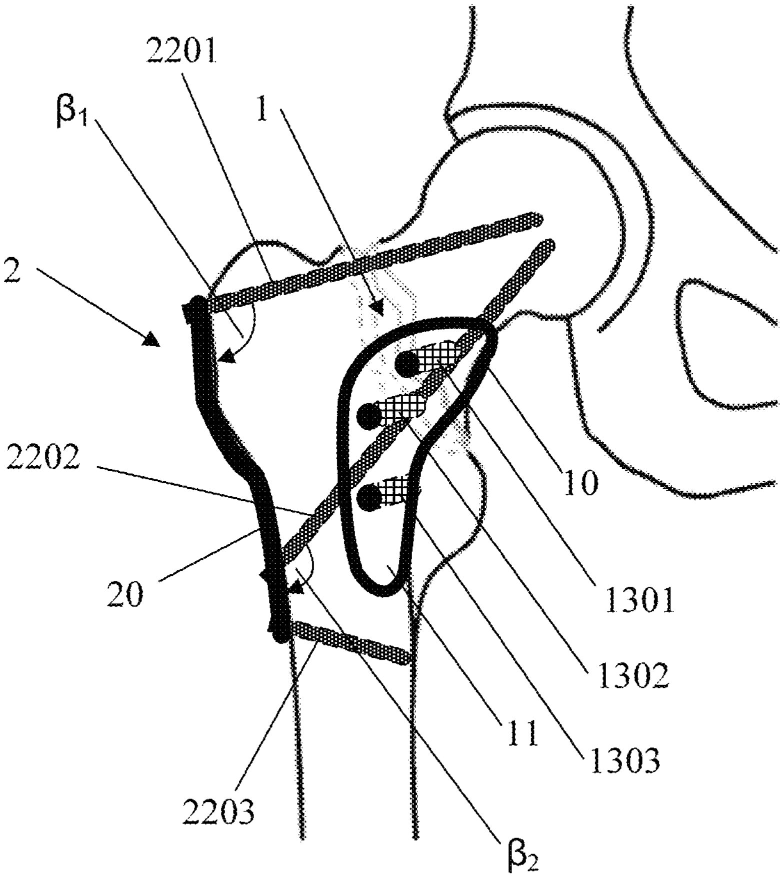

Figure 7

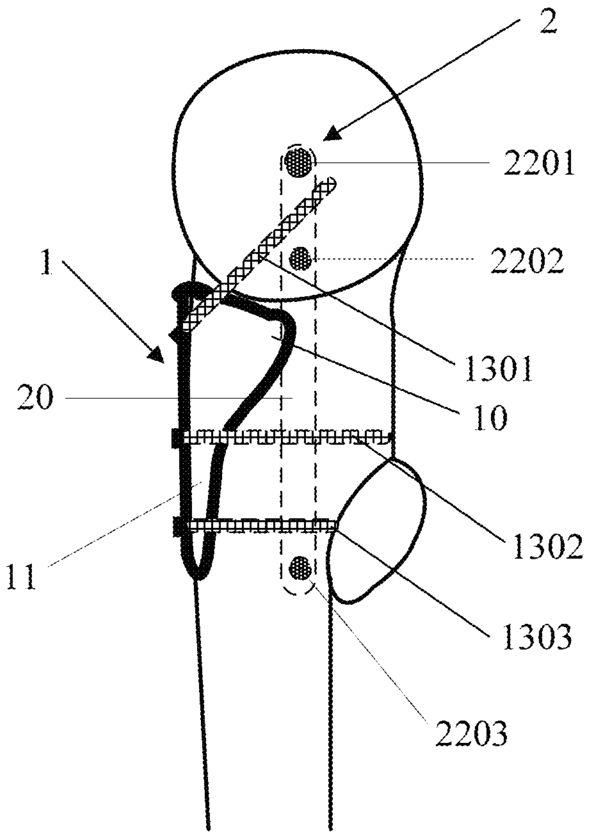

Figure 8

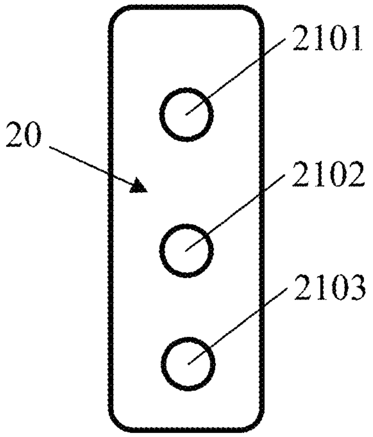

Figure 9

MEDIAL BUTTRESS CONSTRUCT AND A FRACTURE FIXATION DEVICE FOR FEMORAL NECK

This patent application is a U.S. national stage filing under 35 U.S.C. § 371 of International Application No. PCT/CN2016/073541 filed Feb. 4, 2016 (published as WO 2017/132952 on Aug. 10, 2017). The disclosure of the application identified in this paragraph is incorporated herein by reference in its entirety.

TECHNOLOGY FIELD

The present invention relates to the technical field of medical equipment, in particular to a medial buttress construct and a fracture fixation device for femoral neck, which is used for internal fixation of femoral neck fracture to enhance the reliability of fixation after reduction, thereby contributing to fracture healing.

BACKGROUND TECHNIQUE

Femoral neck fracture, as the name implies, is a fracture of the neck of the femur, and usually refers to the fracture between the bottom of the femoral head and the basal part of the femoral neck. The femoral neck fracture is the most common fracture in the hip. In the record of the global statistical hip fracture cases, femoral neck fractures account for about ⅔ of them, and the femoral neck may occur within all ages. Most of femoral neck fractures in a young patient usually are caused by severe violence, such as a traffic accident or a high falling injury. Most of the patients are usually older middle-aged and elderly, and most of femoral neck fractures are caused by careless walking and their buttocks touching the ground. They have pain in the hip and can not stand and walk after injury, and it also may lead to complications such as avascular necrosis of femoral head. In addition, the older patients usually have systemic diseases such as hypertension, heart disease, diabetes and other diseases. Because of the bedridden after injury, a significant decline in resistance and poor rehabilitation ability, it is usually possible to cause complications of several diseases, so the mortality rate of the older patients is higher than that of the general fracture patients. Therefore, the treatment of the femoral neck fracture is one of the important topics in the field of medical technology in department of orthopedics.

From the anatomical and biomechanical characteristics of the femoral neck, it is known that most femoral neck fractures have a distinct shift, and the fracture is unstable. That is, the femoral neck fracture part (and the femoral head) often bears great torsion force and shear force, which affects the stability of the fracture and its reduction, thus affecting the internal fixation effect, and therefore the rate of bone nonunion is higher than other fractures; and because of the special blood supply of the femoral head and the femoral neck, it is easy to cause interruption of blood supply when fracture, thus affect the fracture healing and cause complications such as avascular necrosis of femoral head and collapse of the femoral head. The treatment of femoral neck fracture usually includes conservative treatment and surgical treatment. In view of the higher bone nonunion rate of more than 50% in conservative treatment, active surgical treatment is advocated in modern department of orthopedics. Surgical treatment usually includes closed reduction and internal fixation, open reduction and internal fixation, and artificial joint (prosthesis) replacement. Wherein the closed reduction and internal fixation is less traumatic but has poor fixation effect, and artificial joint (prosthesis) replacement has great trauma and high cost and its fixed effect depends on the quality of artificial joint (prosthesis). Therefore, open reduction and internal fixation is often used, which usually includes three stages: fracture reduction, internal fixation and screw insertion. At present, the internal fixation methods of femoral neck fracture usually use several screws (such as three cannulated screws), and the process of applying the corresponding screw insertion is as follows: the screws are inserted from the bottom of the femoral greater trochanter through the femoral trochanter part (viz. the greater trochanter part), the femoral neck part (via the femoral neck fracture end) to the femoral head part to achieve the effect of fixation for fracture end (that is the broken part of the femoral head). But because the shear force of the fracture end that caused by the longitudinal pressure of the femoral head because of the quality of the femoral head itself and the weight of the patient's body is larger, so the requirement for the fixed position of the screw is very high. If the screw position is not good, it is very easy to lead to screw fixation difficulties, which leads to fixation failure, redislocation of femoral neck fracture part, and then causes bone nonunion; and even if more screws are used or a variety of screw arrangement is used to strengthen the fixation effect, the stability is usually not ideal, the screw's resistance to rotation and torsion is poor, and the femoral head is easily displaced, and then causes femoral neck nonunion, varus displacement of femoral head, femoral neck malunion healing or femoral neck shortening; especially in the majority of middle-aged and elderly patients with osteoporosis, these problems are more prominent and serious. In order to solve these problems, the internal fixation method of femoral neck fracture includes another way, that is, a bone plate is placed on the lateral of the femoral greater trochanter or the basal part of the femoral neck, and the locking nail is inserted into the femoral neck part through the bone plate in order to fix the femoral neck fracture part. However, this method has not solved the problem of the dislocation of the femoral neck fracture part caused by the greater torsion force and shear force suffered by the femoral neck fracture part (and the femoral head).

SUMMARY OF THE INVENTION

The invention aims at the problems in the existing technology that the femoral neck fracture usually fixed by only screws, the screws fixation difficulties and the stability is usually not ideal, and it is easy to produce the femoral head displaced and then lead to the femoral neck nonunion; the method that the bone plate is placed on the lateral of the femoral greater trochanter or the basal part of the femoral neck in order to fix the femoral neck fracture part still doesn't solve the problem of the dislocation of the femoral neck fracture part. For the above problems, the present invention proposes a medial buttress construct for femoral neck which adopts a structure that matches with anatomical parts of femoral neck and upper end of the femoral shaft—a support part and an attachment fixing part. It well supports the femoral neck fracture part, that is to say, it can play a good supporting role for fracture end (viz. the broken femoral head) in the femoral neck fracture inner part through the support part, so that the great torsion force and shear force borne by the femoral neck fracture part are greatly reduced, assisting the fracture part with the accurate positioning, reduction and healing, and the stability after reduc-

tion of the femoral neck fracture is effectively ensured. The present invention also relates to a fracture fixation device for femoral neck.

The technical solution of the present invention is as follows:

A medial buttress construct for femoral neck is characterized in that comprising a support part and an attachment fixing part. The support part and the attachment fixing part are fixedly connected to each other. The support part is used for supporting femoral head and is provided with a structure matching inferior surface of proximal end of the femoral neck, the attachment fixing part is used for fixing the support part and is provided with structures simultaneously matching front side surface of distal end of the femoral neck and front side surface of upper end of femoral shaft, and the attachment fixing part is provided with at least one opening for allowing a screw to pass through on the structure matching the front side surface of the distal end of the femoral neck, and at least one opening for allowing a screw to pass through on the structure matching the front side surface of the upper end of the femoral shaft.

The support part and the attachment fixing part are made by an integrated forming process.

The attachment fixing part is provided with one opening for allowing a screw to pass through on the structure matching the front side surface of the distal end of the femoral neck and two openings for allowing screws to pass through on the structure matching the front side surface of the upper end of the femoral shaft, and the three openings are arranged in a triangle.

The attachment fixing part is provided with one opening for allowing a screw to pass through on the structure matching the front side surface of the distal end of the femoral neck and two openings for allowing screws to pass through on the structure matching the front side surface of the upper end of the femoral shaft, and the three openings are arranged in a line.

A fracture fixation device for femoral neck is characterized in that comprising the medial buttress construct for femoral neck, and also comprises at least two screws for fixing the medial buttress construct for femoral neck and a lateral locking structure for femoral shaft for fixing the femoral head and the femoral shaft. The lateral locking structure for femoral shaft comprises at least two locking nails. Each of the screws is respectively passed through the opening of the structure of the attachment fixing part matching the front side surface of the distal end of the femoral neck and passed through the opening of the structure of the attachment fixing part matching the front side surface of the upper end of the femoral shaft. The locking nail has a length which is larger than minimum distance from facies lateralis of the upper end of femoral shaft to the femoral neck fracture section and then swirled into the femoral head.

The locking nail has three, and the three locking nails arranged in a triangle; and/or the locking nails are arranged in parallel with each other.

The screws are cannulated screws or solid screws; and/or, the locking nails are cannulated nails or solid nails.

The lateral locking structure for femoral shaft also comprises a locking plate which has a structure matching the facies lateralis of the femoral shaft and/or facies lateralis of the greater trochanter. The locking plate is provided with at least one opening for allowing the locking nail to pass through on the structure matching the facies lateralis of the femoral shaft and/or the facies lateralis of the greater trochanter, and the locking nails are passed through the openings of the locking plate.

An angle range of an angle between the locking nail and the locking plate is 89°~130°.

The technical effect of the present invention is as follows:

The present invention relates to a medial buttress construct for femoral neck that comprising the support part and the attachment fixing part with fixed connections and both with specific structures. In particular, the support part is provided with a structure matching the inferior surface (or facies medialis, it can also comprise a part of facies lateralis) of the proximal end of the femoral neck (that is an end of the fracture near the heart), and can provide well support for the femoral neck fracture part, that is, it can play a good supporting role for fracture end (viz. broken femoral head) in the femoral neck inner part through the support part, the attachment fixing part is provided with structures simultaneously matching the front side surface of the distal end of the femoral neck (that is an end of the fracture far away the heart) and the front side surface of the upper end of the femoral shaft, the attachment fixing part and the support part cooperate with each other to achieve effective support and fixation for femoral neck fracture, so that the great torsion force and shear force borne by the femoral neck fracture part are greatly reduced, assisting the fracture part with the accurate positioning, and the stability after reduction of the femoral neck fracture is effectively ensured. The medial buttress construct for femoral neck was implanted through a conventional incision of the anterolateral approach for the thigh (the conventional femoral neck open reduction internal fixation preoperative lateral incision—Watson Jones approach) with a smaller wound, because of the fixed connection between the support part and the attachment fixing part at a certain angle, it can play a good role in supporting femoral head. The attachment fixing part is provided both with at least one opening for allowing a screw to pass through on the structure matching the front side surface of the distal end of the femoral neck and the front side surface of the upper end of the femoral shaft, it can play the fixation effect through the screws from special parts namely the front side surface of the distal end of the femoral neck, specifically, that is to say from the front side surface of the basal area of the femoral neck near the intertrochanter lineae pass through the screw. The intraoperative injury surface and injury degree were less, and the postoperative healing and recovery rate were faster. The medial buttress construct for femoral neck is especially suitable for the femoral neck fracture of the Pauwels angle value $\alpha > 50°$ (Type III/adductor), especially for the situation that needing effectively support femoral head to reduce the torsion force and shear force borne by the femoral neck fracture part, it ensures the blood transport of the fracture part after reduction, improves the healing speed and healing effect of the femoral neck fracture, and greatly reduces the complications such as fracture nonunion and other complications.

The present invention also relates to a fracture fixation device for femoral neck that comprising the medial buttress construct for femoral neck, at least two screws and the lateral locking structure for femoral shaft. The medial buttress construct for femoral neck is used to support the femoral neck fracture location/fracture end (viz. broken femoral head), at least two locking nails of the lateral locking structure for femoral shaft are placed from the facies lateralis of the greater trochanter (and the facies lateralis of the upper end of the femoral shaft) to enter the femoral neck (via the femoral neck fracture end) to the femoral head to further achieve the role of the fixed fracture end; when the lateral locking structure for femoral shaft also includes the locking plate, the locking nail firstly passes through the

opening of the locking plate and then incises from the facies lateralis of the greater trochanter (and the facies lateralis of the upper end of the femoral shaft) to enter the femoral neck (via the femoral neck fracture end) to the femoral head, the locking plate can further strengthen the connection stability of the femoral shaft and femoral head, and improve the healing rate of the femoral neck fracture. The lateral locking structure for femoral shaft combined with the medial buttress construct for femoral neck to achieve the doubling effective support and stable fixation effect; and the whole fracture fixation device for femoral neck can be implanted through a conventional incision of the anterolateral approach for the thigh (the conventional femoral neck open reduction internal fixation preoperative lateral incision—Watson Jones approach), in other words, both the lateral locking structure for femoral shaft and the medial buttress construct for femoral neck can be implanted through the same conventional incision of the anterolateral approach for the thigh without increasing the existing technical injury surface, injury degree and bleeding volume. The postoperative healing speed is faster and the healing rate is high.

DESCRIPTION OF THE DRAWINGS

FIG. **1** is a positive view of a preferred structure of the medial buttress construct for femoral neck of the present invention.

FIG. **2** is the side view (left 45°) of FIG. **1**.

FIG. **3** is a positive view of another preferred structure of the medial buttress construct for femoral neck of the present invention.

FIG. **4** is a structural schematic diagram of use state of the preferred medial buttress construct for femoral neck of the present invention.

FIG. **5** is a schematic diagram of the Pauwels angle.

FIG. **6** is a structural schematic diagram of use state of the preferred fracture fixation device for femoral neck of the present invention.

FIG. **7** is a structural schematic diagram of use state of another preferred fracture fixation device for femoral neck of the present invention.

FIG. **8** is the right view of FIG. **7**.

FIG. **9** is a positive view of the preferred structure of the locking plate of the present invention.

The numbers in the diagram are listed as follows:

1—medial buttress construct for femoral neck; **10**—support part; **11**—attachment fixing part; **1201**—first screw opening; **1202**—second screw opening; **1203**—third screw opening; **1301**—first screw; **1302**—second screw; **1303**—third screw; **2**—lateral locking structure for femoral shaft; **20**—locking plate; **2101**—first locking nail opening; **2102**—second locking nail opening; **2103**—third locking nail opening; **2201**—first locking nail; **2202**—second locking nail; **2203**—third locking nail;

α—Pauwels angle; β_1—an angle between the first locking nail and the locking plate; $\beta 2$—an angle between the second locking nail and the locking plate.

DESCRIPTION OF THE PREFERRED EMBODIMENTS

The present invention will be described below with reference to the accompanying drawings.

The present invention relates to a medial buttress construct for femoral neck that comprising the support part and an attachment fixing part, and the support part and the attachment fixing part are fixedly connected to each other; the support part is used for supporting femoral head and is provided with a structure matching inferior surface of proximal end of the femoral neck, the attachment fixing part is used for fixing the support part and is provided with structures simultaneously matching front side surface of distal end of the femoral neck and front side surface of upper end of femoral shaft, or understood as that the attachment fixing part is simultaneously provided with a structure matching front side surface of distal end of the femoral neck and a structure matching front side surface of upper end of the femoral shaft, and the attachment fixing part is provided with at least one opening for allowing a screw to pass through on the structure matching the front side surface of distal end of the femoral neck, and at least one opening for allowing a screw to pass through on the structure matching the front side surface of the upper end of the femoral shaft.

Preferably, the support part and the attachment fixing part can be made by seamless connection technology or made by an integrated forming process, and can be made of stainless steel or other metal alloy materials. The specific making process and making material can be set according to the actual application, including, but not limited to the above limits.

A preferred structure of the medial buttress construct for femoral neck **1** proposed by the present invention is shown in FIGS. **1** and **2**, wherein FIG. **1** is a positive view and FIG. **2** is a 45° left side view, the medial buttress construct for femoral neck **1** comprises a support part **10** and an attachment fixing part **11**, the support part **10** and the attachment fixing part **11** are fixedly connected to each other, as shown by a dotted line, the support part **10** is provided with a structure matching the inferior surface (or facies medialis, it can also comprise a part of facies lateralis) of the proximal end of the femoral neck (that is an end of the fracture near the heart), (that is to say, the support **10** has a curved/arc structure matching with the facies medialis of proximal end of the femoral neck and a corresponding facies medialis, whose stereoscopic effects are not shown in FIGS. **1** and **2**), the attachment fixing part **11** is a part other than the support part **10**, the attachment fixing part **11** is simultaneously provided with structures matching the front side surface of the distal end of the femoral neck (that is an end of the fracture far away the heart)(that is to say, the attachment fixing part **11** has a curved/arc structure matching with the front side surface of the basal area of the femoral neck near the intertrochanter lineae and the corresponding facies medialis, whose stereoscopic effects is not shown in FIGS. **1** and **2**) and structures matching the front side surface of the upper end of the femoral shaft (that is to say, the attachment fixing part **11** has a curved/arc structure matching the front side surface of the upper end of the femoral shaft and the corresponding facies medialis, whose stereoscopic effects are not shown in FIGS. **1** and **2**), the attachment fixing part **11** is provided with two openings for allowing screws to pass through: a first screw opening **1201** and a second screw opening **1202**, and the location of the first screw opening **1201** is specially set on the structure of the attachment fixing part **11** matching the front side surface of the distal end of the femoral neck for a special fixed effect, namely, through the attachment fixing part **11** fixed the support part **10** and then achieve the control of femoral head part and fixed effect. The second screw opening **1202** is set on the structure of the attachment fixing part **11** matching the front side surface of the upper end of the femoral shaft. Preferably, the support part **10** and the attachment fixing part **11** can be made by an integrated forming process, and the size and details of the support part **10** and the attachment fixing part

11 can be set reasonably in accordance with the specific anatomical data of the patient's individual and the combination of historical experience data and experimental data. The medial buttress construct for femoral neck **1** proposed by the present invention adopts a structure that comprising the integrated support part **10** and the attachment fixing part **11**, the support part **10** adopts a structure that matching the anatomical part of the proximal end of femoral neck (below the femoral head), can provide well support for the femoral neck fracture part, that is, it can play a good supporting role for the femoral neck fracture part/fracture end (viz. the broken femoral head) in the femoral neck inner part through the support part, so that the great torsion force and shear force borne by the femoral neck fracture part are greatly reduced, assisting the fracture part with the accurate positioning, and the stability after reduction of the femoral neck fracture is effectively ensured; the attachment fixing part **11** adopts a structure that matching the anatomical parts of the front side surface of distal end of femoral neck and the front side surface of upper end of femoral shaft, reasonable use of human anatomy data structure can achieve the positioning and fixation on the basal area of femoral neck, and can play a good attachment, fixation effect, further guarantees assisting the fracture part with the accurate positioning, reduction and healing; and effectively solved the problem of the dislocation of the femoral neck fracture part caused by the greater torsion force and shear force suffered by the femoral neck fracture part (and the femoral head).

The positive view of another preferred structure of the medial buttress construct for femoral neck **1** proposed by the present invention is shown in FIG. **3**, wherein, the medial buttress construct for femoral neck **1** similarly comprises a support part **10** and an attachment fixing part **11** with fixed connections and both with specific structures, the difference between the embodiments shown in FIGS. **1** and **2** is that the attachment fixing part **11** is provided with three openings for allowing screws to pass through in the embodiments shown in FIG. **3**: a first screw opening **1201**, a second screw opening **1202** and a third screw opening **1203**, and the location of the first screw opening **1201** is specially set on the structure of the attachment fixing part **11** matching the front side surface of the distal end of the femoral neck for a special fixed effect, namely, through the attachment fixing part **11** fixed the support part **10** and then achieve the control of femoral head part and fixed effect. Both the second screw opening **1202** and the third screw opening **1203** are set on the structure of the attachment fixing part **11** matching the front side surface of the upper end of the femoral shaft. In addition, as shown in FIG. **3**, the first screw opening **1201**, the second screw opening **1202** and the third screw opening **1203** can be arranged in a triangle, of course, also can be arranged in a line, the arrangement shape can be set arbitrarily only to ensure that it can play an effective fixing effect and to ensure as far as possible in the actual use of patients with less damage; and the attachment fixing part **11** also can be provided with more than three openings for allowing screws to pass through, the numbers and the arrangement shape can be set according to the actual needs only to ensure that it can play an effective fixing effect and to ensure as far as possible in the actual use of patients with less damage.

The operational principle and use method of the medial buttress construct for femoral neck **1** proposed by the present invention are as follows:

The structural schematic diagram of use state of the preferred medial buttress construct for femoral neck **1** of the present invention is shown in FIG. **4**, in the process of actual use, the medial buttress construct for femoral neck **1** as shown in FIG. **1**, FIG. **2** and FIG. **3** was implanted through a conventional incision of the anterolateral approach for the thigh (the conventional femoral neck open reduction internal fixation preoperative lateral incision—Watson Jones approach), the support part **10** is placed below the femoral head, the facies medialis and the front side surface of the proximal end of the femoral neck, the facies medialis of the support part **10** is closely connected with the facies medialis of the proximal end of the femoral neck and the front side surface of the proximal end of the femoral neck, which may be part of it (the facies medialis of the support part **10** can also be extended to close contact with part of the lower side surface of the femoral head, that is the anterior part of the support part **10** can be extended to the lower part of the femoral head), and plays a good supporting role for supporting femoral head. The implantation position of the attachment fixing part **11** is from the basal part of the femoral neck to the upper part of the femoral shaft, and the facies medialis of the upper part of the attachment fixing part **11** is closely connected with the basal part of the femoral neck that is the front side surface of the distal end of the femoral neck, and the facies medialis of the middle and lower part of the attachment fixing part **11** is closely connected with the front side surface of the upper end of the femoral shaft. The support part **10** and the attachment fixing part **11** cooperate with each other to prefectly achieve effective support and fixation for femoral neck fracture, so that the great torsion force and shear force borne by the femoral neck fracture part are greatly reduced, assisting the fracture part with the accurate positioning, and the stability after reduction of the femoral neck fracture is effectively ensured. The embodiment shown in FIG. **4** adopts the medial buttress construct for femoral neck **1** that provided with three openings shown in FIG. **3**, the first screw opening **1201**, the second screw opening **1202** and the third screw opening **1203** of the attachment fixing part **11** are used to penetrate a first screw **1301**, a second screw **1302** and a third screw **1303** respectively to play a role in fixing the medial buttress construct for femoral neck **1**, and the first screw opening **1201** especially can play the fixation effect through the first screw **1301** from special parts namely the front side surface of the distal end of the femoral neck, and the first screw **1301** through the first screw opening **1201** can be penetrated by appropriate angle according to the anatomical data and structure of the front side surface of the distal end of the femoral neck, and the second screw **1302** and the third screw **1303** through the second screw opening **1202** and the third screw opening **1203** can be inserted from the front side surface of the upper end of the femoral shaft at a appropriate angle, without increasing intraoperative damage surface, injury degree and bleeding volume of the existing technology, and the injury surface and degree of injury are usually smaller and the amount of bleeding is less, and postoperrative faster healing and recovery. In addition, as the embodiment shown in FIG. **4**, the first screw **1301**, the second screw **1302** and the third screw **1303** respectively cut in a certain angle from the front side surface of the distal end of the femoral neck and the front side surface of the upper end of the femoral shaft, and the screw body parts of the screws in the "dotted line frame and the inner filling vertical mesh shape" shown in FIG. **4** respectively represent the projections of the screw body parts of the first screw **1301**, the second screw **1302** and the third screw **1303** on the vertical plane.

In addition, the medial buttress construct for femoral neck **1** proposed by the present invention usually suitable for the femoral neck fracture of the Pauwels angle (that is α angle)

value α>50° (Type III/adductor) as shown in FIG. **5**. As shown in FIG. **5**, the Pauwels angle usually refers to an angle between the fracture line and the connection line of two iliac crest when the femoral neck fracture occurs, and generally includes three types: Type I: α<30°, in order to stabilize the fracture, there are many intercalation, small shear, more stable, higher healing rate, often can be used continuous traction treatment, but if it is not handled properly, displacement can occur, and it can be shifted to unstable type, that is type III; Type II: 30°≤α≤50°, the stability of fracture is between Type I and Type III; Type III: α>50°, unstable fracture, multiple displacement, large shear force, difficult fixation, low healing rate. From the above, the bigger Pauwels angle, with the greater the shear fracture, and for more unstable fracture, usually needs internal fixation. The medial buttress construct for femoral neck **1** proposed by the present invention focuses on the defects and deficiencies in the internal fixation of the femoral neck fracture reduction that due to the greater torsional force and shear force borne by the femoral neck fracture (and the femoral head) resulting in the dislocation of the fracture of the femoral neck, the poor stability of the existing internal fixation resulting in the femoral neck nonunion. Therefore, it is preferable to apply the femoral neck fracture with Pauwels angle value α>50° (that is type III), it can well supports the femoral neck fracture part, especially the proximal end, so that the great torsion force and shear force borne by the femoral neck fracture part are greatly reduced, assisting the fracture part with the accurate positioning, and the stability and heal effect after reduction of the femoral neck fracture are effectively ensured.

The present invention also relates to a fracture fixation device for femoral neck that comprising the medial buttress construct for femoral neck, the lateral locking structure for femoral shaft and at least two screws, wherein, the medial buttress construct for femoral neck adopts the medial buttress construct for femoral neck **1** as shown in FIGS. **1**-**4**, the screws is used to fix the medial buttress construct for femoral neck and each of the screws is respectively passed through the opening of the structure of the attachment fixing part matching the front side surface of the distal end of the femoral neck and passed through the opening of the structure of the attachment fixing part matching the front side surface of the upper end of the femoral shaft; the lateral locking structure for femoral shaft is used to fix the femoral head and the femoral shaft, and the lateral locking structure for femoral shaft comprises at least two locking nails, the locking nail has a length which is larger than minimum distance from facies lateralis of the upper end of femoral shaft to the femoral neck fracture section and then swirled into the femoral head, and it was preferred to the 1 cm of the subchondral bone of the femoral head.

The lateral locking structure for femoral shaft preferably also comprises a locking plate, the locking plate has a structure matching the facies lateralis of the femoral shaft and/or facies lateralis of the greater trochanter; the locking plate is provided with at least one opening for allowing the locking nail to pass through on the structure matching the facies lateralis of the femoral shaft and/or the facies lateralis of the greater trochanter, and the locking nails are passed through the openings of the locking plate. The structure including the locking plate and the locking nails forms the lateral locking structure for femoral shaft.

The structural schematic diagram of use state of the preferred fracture fixation device for femoral neck of the present invention is shown in FIG. **6**, comprising the medial buttress construct for femoral neck **1**, three screws and two locking nails, wherein, the three screws are the first screw **1301**, the second screw **1302** and the third screw **1303**, each screws respectively pass through the openings of the attachment fixing part **11** (that is the first screw opening **1201**, the second screw opening **1202**, the third screw opening **1203**, not shown in FIG. **6**), the first screw **1301** passes through the opening on the structure of the attachment fixing part **11** matching the front side surface of the distal end of the femoral neck (that is the first screw opening **1201**), the second screw **1302** and the third screw **1303** pass through the openings on the structure of the attachment fixing part **11** matching the front side surface of the upper end of the femoral shaft (that is the second screw opening **1202** and the third screw opening **1203**); the two locking nails are the first locking nail **2201** and the second locking nail **2202**, each of the locking nails has a length which is larger than minimum distance from the facies lateralis of greater trochanter to the femoral neck fracture section. Preferably, the number of openings on the attachment fixing part **11** and the number of corresponding screws can be set more, and the screws can be arranged in parallel or a part or all of them arranged in a certain angle, and between the screws and the attachment fixing part **11** can also be arranged in a certain angle. The screws can be selected for cannulated screw or solid screw, the number of screws, the arrangement shape of screw, and the angle between the screw and the attachment fixing part **11**, all can be set according to the actual needs, only to ensure that it can play an effective fixing effect and to ensure as far as possible in the actual use of patients with less damage; the locking nails may be selected as cannulated nail or solid nail, preferably anti-rotation cannulated or solid nails, off-axis cannulated or solid nails that can resist torsional force and shear force, the locking body diameter range of the locking nail can be 3.5 mm~7.5 mm, preferably as in the example shown in FIG. **6**, the locking nail body diameter of the first locking nail **2201** is 6.5 mm, the locking nail body diameter of the second locking nail **2202** is 4.5 mm, and the locking nail body front part of each of locking nails can also be threaded to further strengthen the locking effect, and the number of locking nails can be more, and the locking nails can be arranged in parallel or a part or all of them arranged in a certain angle, and the locking nails can also be set in a certain angle with the facies lateralis of the greater trochanter and with the facies lateralis of upper end of the femoral shaft (that is the first locking nail **2201** can be set in a certain angle with the facies lateralis of the greater trochanter; the second locking nail **2202** can be set in a certain angle with the facies lateralis of upper end of the femoral shaft; the third locking nail **2203** can be set in a certain angle from the facies lateralis of the upper end of the femoral shaft to the upper end of the femoral shaft), through reasonable layout, the locking nails bear different stresses respectively, to achieve that assisting the fracture part with the accurate positioning and stable reduction fixation; adjusting the position and angle of the locking nails cut through the fracture section of the femoral neck to improve the fixed firmness and stability, the number of locking nails, the arrangement shape of the locking nails, the angle between the locking nails and the facies lateralis of the greater trochanter and the facies lateralis of the femoral shaft, and the position and angle of the locking nail cut through the fracture section of the femoral neck, all can be set according to the actual needs, only to ensure that it can play an effective fixing effect and to ensure as far as possible in the actual use of patients with less damage.

The structural schematic diagram of use state of another preferred fracture fixation device for femoral neck of the

present invention is shown in FIG. **7** and FIG. **8**, wherein, FIG. **7** is positive view, FIG. **8** is the right view, comprising the medial buttress construct for femoral neck **1**, three screws and the lateral locking structure for femoral shaft **2**, wherein, the three screws are the first screw **1301**, the second screw **1302** and the third screw **1303**, each of screws respectively passes through the opening of the attachment fixing part **11** (that is the first screw opening **1201**, the second screw opening **1202**, the third screw opening **1203**, not shown in FIG. **7** and FIG. **8**), and the lateral locking structure for femoral shaft **2** comprises a locking plate **20** and three locking nails, the three locking nails are the first locking nail **2201**, the second locking nail **2202** and the third locking nail **2203**, each of the locking nails has a length which is larger than minimum distance from the facies lateralis of greater trochanter to the femoral neck fracture section; the type, structure and size setting of the locking nails can refer to the embodiment shown in FIG. **6** above; in addition, as shown in FIG. **7**, the locking plate **20** is provided with a structure matching with the facies lateralis of the greater trochanter and the facies lateralis of the upper end of the femoral shaft, and the locking plate can be made of stainless steel or other metal alloy materials and made by an integrated forming process, the concrete structure shape, making process and making material of the locking plate **20** can be set according to the actual application, including, but not limited to above limits; the locking plate **20** have three openings for allowing the locking nails to pass through, that is the first locking nail opening **2101**, the second locking nail opening **2102**, the third locking nail opening **2103** as shown in FIG. **9**, the locking plate **20** preferably uses a simple rectangular structure, the locking strews respectively pass through the openings of the locking plate for fixing the fracture section (viz. broken femoral head), the first locking nail **2201** passes the femoral head part through the facies lateralis of the greater trochanter via femoral neck fracture section, the second locking nail **2202** pass the femoral head part through the facies lateralis of the upper end of the femoral shaft via femoral neck fracture section the first locking nail **2201** and the second locking nail **2202** and the locking plate **20** can be set in a certain angle β, and the angle range of the β can be 89°~130° (corresponding to the angle value of the femoral neck fracture Pauwels angle $\alpha > 50°$, that is Type III), wherein, the angle between the first locking nail **2201** and the locking plate **20** is preferred to be 95°, the angle β_2 between the second locking nail **2202** and the locking plate **20** is preferred to be 120°, and it can be set according the actual solution, only to ensure that it can play an effective fixing effect and to ensure as far as possible in the actual use of patients with less damage, by the combination of the locking nails (the first locking nail **2201** and the second locking nail **2202**) and the locking plate **20**, it can achieves optimal fixed stability; the third locking nail **2203** enters from the facies lateralis of the upper end of the femoral shaft to the upper end of the femoral shaft), and between the third locking nail **2203** and the locking plate **20** can be set in a certain angle, and the angle range can be 45°~85°, preferably 60°, and it can be set according the actual solution, only to ensure that it can play an effective fixing effect and to ensure as far as possible in the actual use of patients with less damage, the combination of the three locking nails and the locking plate **20** can further enhance the optimum fixed stability to the maximum extent.

In addition, as the embodiments shown in FIG. **6**, FIG. **7** and FIG. **8**, the first screw **1301**, the second screw **1302** and the third screw **1303** respectively cut in a certain angle from the front side surface of the distal end of the femoral neck and the front side surface of the upper end of the femoral shaft, and the screw body parts of the screws in the "dotted line frame and the inner filling vertical mesh shape" shown in FIG. **6**, FIG. **7** and FIG. **8**, respectively represent the projection of the screw body part of the first screw **1301**, the second screw **1302** and the third screw **1303** on the vertical plane. The first locking nail **2201**, the second locking nail **2202** and the third locking nail **2203** respectively cut in a certain angle from the facies lateralis of the greater trochanter and the facies lateralis of the upper end of the femoral shaft, and the locking nail body parts of the locking nails in the "dotted line frame and the inner filling vertical mesh shape" shown in FIG. **6**, FIG. **7** and FIG. **8**, respectively represent the projection of the locking nail body part of the first locking nail **2201**, the second locking nail **2202** and the third locking nail **2203** on the vertical plane. Moreover, the screw body part of each screw is not in a plane with the locking nail part of each locking nail, and do not touch each other.

The operational principle and use method of the fracture fixation device for femoral neck proposed by the present invention are as follows:

As shown in FIG. **6**, FIG. **7** and FIG. **8**, in the process of actual use, the fracture fixation device for femoral neck proposed by the present invention can absolutely implanted through a conventional incision of the anterolateral approach for the thigh (the conventional femoral neck open reduction internal fixation preoperative lateral incision—Watson Jones approach), in other words, both the lateral locking structure for femoral shaft **2** and the medial buttress construct for femoral neck **1** can be implanted through the same conventional incision of the anterolateral approach for the thigh (the conventional femoral neck open reduction internal fixation preoperative lateral incision—Watson Jones approach), only one incision is needed to achieve the insertion of the fracture fixation device for femoral neck proposed by the invention, and the injury surface and degree of injury are usually smaller and the amount of bleeding is less, and postoperrative healing and recovery are faster, the operational principle and use method of the medial buttress construct for femoral neck **1** proposed showed in FIG. **1** and FIG. **2** are as described above. Moreover, the lateral locking structure for femoral neck **2** adopts at least two locking nails from the facies lateralis of the greater trochanter of the femoral (and the facies lateralis of the upper end of the femoral shaft), through the femoral greater trochanter part (and the upper end of the femoral shaft), the femoral neck part (viz. femoral neck fracture section), to femoral head part, to play a role for fixing the femoral fracture end (viz. broken femoral head), when the lateral locking structure for femoral shaft **2** further adopts the locking plate, the locking plate **20** is closely connected with the facies lateralis of the greater trochanter and the facies lateralis of the upper end of the femoral shaft, the locking nails firstly passes through the openings of the locking plate **20** and then incises from the facies lateralis of the greater trochanter and the facies lateralis of the upper end of the femoral shaft to enter the femoral head and the upper end of femoral shaft for fixing the femoral fracture end (viz. the broken femoral head). The combination of the lateral locking structure for femoral shaft **2** and the medial buttress construct for femoral neck **1** realizes the fixed effect of 1+1>2, further strengthens the effective support and fixation for the femoral neck fracture, reduces the great torsion force and shear force borne by the femoral neck fracture part, assists the fracture part with the accurate positioning, and the stability after reduction of the femoral neck fracture are effectively ensured.

It should be noted that the above-described embodiments may make those skilled in the art more fully understand the invention, but do not limit the invention in any way. Therefore, although the present specification has been described in detail with reference to the accompanying drawings and embodiments, it should be understood by those skilled in the art that the invention can still be modified or equivalently replaced. In short, all technical solutions and improvements that do not deviate from the spirit and scope of the present invention shall all be covered by the protection scope of the present patent.

What is claimed is:

1. A medial buttress construct for femoral neck comprising a support part and an attachment fixing part, the support part and the attachment fixing part are fixedly connected to each other, the support part is configured to be used for supporting a femoral head and includes a structure matching an inferior surface of a proximal end of the femoral neck thereby allowing the support part to be positionable in close contact with the inferior surface of the proximal end of the femoral neck, the attachment fixing part is configured to be used for fixing the support part and includes structures simultaneously matching an anterior surface of a distal end of the femoral neck and an anterior surface of an upper end of a femoral shaft thereby allowing the attachment fixing part to be positionable in close contact with the anterior surface of the distal end of the femoral neck and the anterior surface of the upper end of the femoral shaft at the same time, and the attachment fixing part includes at least one opening for allowing a screw to pass through the structure matching the anterior surface of the distal end of the femoral neck and penetrate into the anterior surface of the distal end of the femoral neck, and at least one opening for allowing a screw to pass through the structure matching the anterior surface of the upper end of the femoral shaft and penetrate into the anterior surface of the upper end of the femoral shaft.

2. The medial buttress construct for femoral neck according to claim **1**, wherein the support part and the attachment fixing part are made by an integrated forming process.

3. The medial buttress construct for femoral neck according to claim **2**, wherein the attachment fixing part includes one opening for allowing a screw to pass through the structure matching the anterior surface of the distal end of the femoral neck and penetrate into the anterior surface of the distal end of the femoral neck, and two openings for allowing screws to pass through the structure matching the anterior surface of the upper end of the femoral shaft and penetrate into the anterior surface of the upper end of the femoral shaft, and wherein the one opening and the two openings are arranged in a triangle.

4. The medial buttress construct for femoral neck according to claim **2**, wherein the attachment fixing part includes one opening for allowing a screw to pass through the structure matching the anterior surface of the distal end of the femoral neck and penetrate into the anterior surface of the distal end of the femoral neck, and two openings for allowing screws to pass through the structure matching the anterior surface of the upper end of the femoral shaft and penetrate into the anterior surface of the upper end of the femoral shaft, and wherein the one opening and the two openings are arranged in a line.

5. The medial buttress construct for femoral neck according to claim **1**, wherein the attachment fixing part includes one opening for allowing a screw to pass through the structure matching the anterior surface of the distal end of the femoral neck and penetrate into the anterior surface of the distal end of the femoral neck; and two openings for allowing screws to pass through the structure matching the anterior surface of the upper end of the femoral shaft and penetrate into the anterior surface of the upper end of the femoral shaft, and wherein the one opening and the two openings are arranged in a triangle.

6. The medial buttress construct for femoral neck according to claim **1**, wherein the attachment fixing part includes one opening for allowing a screw to pass through the structure matching the anterior surface of the distal end of the femoral neck and penetrate into the anterior surface of the distal end of the femoral neck; and two openings for allowing screws to pass through the structure matching the anterior surface of the upper end of the femoral shaft and penetrate into the anterior surface of the upper end of the femoral shaft, and wherein the one opening and the two openings are arranged in a line.

7. A fracture fixation device for femoral neck comprising the medial buttress construct for femoral neck according to claim **1** and at least two screws for fixing the medial buttress construct for femoral neck and a lateral locking structure for femoral shaft for fixing the femoral head and the femoral shaft, the lateral locking structure for femoral shaft comprises at least two locking nails, each of the at least two screws is respectively passed through the opening of the structure of the attachment fixing part matching the anterior surface of the distal end of the femoral neck and passed through the opening of the structure of the attachment fixing part matching the anterior surface of the upper end of the femoral shaft, the at least two locking nails each have a length which is larger than minimum distance from the facies lateralis of the upper end of the femoral shaft to the femoral neck fracture section and is configured to be swirled into the femoral head.

8. The fracture fixation device for femoral neck according to claim **7**, wherein the at least two locking nails comprise three locking nails arranged in a triangle or arranged in parallel with each other.

9. The fracture fixation device for femoral neck according to claim **8**, wherein the at least two screws are cannulated screws or solid screws and/or the at least two locking nails are cannulated nails or solid nails.

10. The fracture fixation device for femoral neck according to claim **9**, wherein the lateral locking structure for femoral shaft comprises a locking plate having a structure matching the facies lateralis of the femoral shaft and/or the facies lateralis of the greater trochanter, the locking plate includes openings for allowing the at least two locking nails to pass through the structure matching the facies lateralis of the femoral shaft and/or the facies lateralis of the greater trochanter, and the at least two locking nails are passed through the openings of the locking plate.

11. The fracture fixation device for femoral neck according to claim **10**, wherein an angle range of an angle between at least one of the at least two locking nails and the locking plate is 89°-130°.

12. The fracture fixation device for femoral neck according to claim **7**, wherein the at least two screws are cannulated screws or solid screws; and/or wherein the at least two locking nails are cannulated nails or solid nails.

13. The fracture fixation device for femoral neck according to claim **12**, wherein the lateral locking structure for femoral shaft also comprises a locking plate which has a structure matching the facies lateralis of the femoral shaft and/or the facies lateralis of the greater trochanter, the locking plate includes openings for allowing the at least two locking nails to pass through the structure matching the

facies lateralis of the femoral shaft and/or the facies lateralis of the greater trochanter, and the at least two locking nails are passed through the openings of the locking plate.

14. The fracture fixation device for femoral neck according to claim **13**, wherein an angle range of an angle between at least one of the at least two locking nails and the locking plate is 89°-130°.

15. The fracture fixation device for femoral neck according to claim **7**, wherein the medial buttress construct, the at least two screws, and the lateral locking structure are configured to be implanted through a same single conventional incision.

16. The medial buttress construct for femoral neck according to claim **1**, wherein the support part is configured such that a facies medialis of the support part is configured to be closely connected with the inferior surface of the proximal end of the femoral neck and the anterior surface of the proximal end of the femoral neck.

17. The medial buttress construct for femoral neck according to claim **1**, wherein the support part's structure matching the inferior surface of the proximal end of the femoral neck is configured such that the support part is positionable in direct contact with a surface of a bone defining the inferior surface of the proximal end of the femoral neck.

18. The medial buttress construct for femoral neck according to claim **1**, wherein the support part is configured to be positionable in between a ligamentous hip capsule and the inferior surface of the proximal end of the femoral neck.

19. The medial buttress construct for femoral neck according to claim **1**, wherein:

the support part's structure matching the inferior surface of the proximal end of the femoral neck comprises a curved/arc structure matching with the proximal end of the femoral neck and corresponding facies medialis;

the attachment fixing part's structure matching the anterior surface of the distal end of the femoral neck comprises a curved/arc structure matching with the anterior surface of the basal area of the femoral neck near the intertrochanter lineae and corresponding facies medialis; and

the attachment fixing part's structure matching the anterior surface of the upper end of the femoral shaft comprises a curved/arc structure matching the anterior surface of the upper end of the femoral shaft and corresponding facies medialis.

20. The medial buttress construct for femoral neck according to claim **1**, wherein the attachment part is configured to be directly alignable with an anterior femur side.

* * * * *

UNITED ST
DEP
